# Clinical Roles in the Medical Communications Centre: A Rapid Scoping Review

**DOI:** 10.7759/cureus.39441

**Published:** 2023-05-24

**Authors:** Jennifer A Greene, Judah Goldstein, Jeffrey Stirling, Janel M Swain, Ryan Brown, Jennifer McVey, Alix Carter

**Affiliations:** 1 Emergency Medicine, Dalhousie University, Halifax, CAN; 2 Emergency Health Services, Nova Scotia, Dartmouth, CAN; 3 Interprofessional Practice and Learning, Nova Scotia Health, Sydney, CAN

**Keywords:** secondary triage, medical communications centre, emergency telecare, emergency advice line, clinical support line

## Abstract

In recent years, 911 call volumes have increased, and emergency medical services (EMS) are routinely stretched beyond capacity. To better match resources with patient needs, some EMS systems have integrated clinician roles into the emergency medical communications centre (MCC). Our objective was to explore the nature and scope of clinical roles in emergency MCCs. Using a rapid scoping review methodology, we searched PubMed for studies related to any clinical role employed within an emergency MCC. We accepted reviews, experimental and observational designs, as well as expert opinions. Studies reporting on dispatcher recognition and pre-arrival instructions were excluded. Title and abstract screening were conducted by a single reviewer, included studies were verified by two reviewers, and data extraction was completed in duplicate, all using Covidence review software. The level of evidence was assessed using the prehospital evidence-based practice (PEP) scale. The protocol was registered in Open Science Framework (10.17605/OSF.IO/NX4T8).

Our search yielded 1071 titles, and four were added from other sources; 44 studies were reviewed at the full-text stage and 31 were included. The included studies were published from 2002 to 2022 and represent 17 countries. Studies meeting inclusion criteria consisted of level I (n=4, 11%), II (n=13, 37%), and III (N=6, 17%) methodologies, as well as 12 other studies (34%) with qualitative or other designs. Most of the included studies reported systems that employ nurses in the MCC (n=29, 83%). Twelve (34%) studies reported on the inclusion of paramedics in the MCC, and five (14%) reported physician involvement. The roles of these clinicians chiefly consisted of triage (n=25, 71%), advice (n=20, 57%), referral to non-emergency care (n=14, 40%), and peer-to-peer consulting (n=2, 4%). Alternative dispositions (as opposed to emergency ambulance transport) for low acuity callers included self-care, as well as referral to a general practitioner, pharmacist, or other outreach programs. There is a wide range of literature reporting on clinical roles integrated within MCCs. Our findings revealed that MCC nurses, physicians, and paramedics assist substantively with triage, advice, and referrals to better match resources to patient needs, with or without the requirement for ambulance dispatch.

## Introduction and background

In recent years, healthcare systems have been stretched by increasing patient volume, staffing shortages, low access to primary care, and emergency department (ED) closures. These challenges have led in part to a dramatic increase in 911 call volumes resulting in major operational strains [1-5]. This crisis has led to a growing interest in developing alternative arrangements for patients following 911 contacts. Non-dispatch of paramedic units for select low-acuity callers, treat and release pathways, and emergency medical services (EMS) system-initiated non-transport with an alternative disposition/follow-up plan are all areas of growing interest for contemporary ground ambulance systems [3]. The aim is to “get the right patient to the right place at the right time” [6-8].

In 2014, approximately 20% of all 911 calls, locally, in Nova Scotia (NS) resulted in a non-transport disposition [9]. Provincial continuous quality improvement data indicate that the number of non-transports has increased to approximately 35% in recent years [10]. This is consistent with the international published experiences, indicating global non-conveyance rates ranging from 12 to 51% [11-15]. A recent Swedish study reported that half of the non-conveyed patients were able to be referred to self-care [13].

The traditionally employed approach of sending an ambulance to all 911 callers and transporting all patients to the ED does not always result in providing the right care for the right patient, at the right time and place. In order to better match resources with the needs of the patient, some EMS services have integrated clinician roles within their medical communications centres (MCCs). The MCC is where 911 calls that are determined to require medical attention are received and from where resources are dispatched. Some examples of clinical roles include providing advice to patients over the phone, secondary (communications centre level) triage, or arranging access to the most appropriate resource instead of deploying a paramedic crew. The National Health Service in the United Kingdom has employed this model since the 1990s [16]. This is stated to be a key component of their emergency system ensuring “the right advice, in the right place, at the first point of contact” [16].

In this review, our primary objective was to describe clinical roles developed and studied within MCCs. The secondary objectives were to describe safety and efficacy outcomes reported from the implementation of these roles.

This work was presented as an abstract at the Paramedic Association of Canada Expo on September 8, 2022, at Paramedicine Research Day on May 26, 2022, at the Dalhousie University Emergency Medicine Research Series on April 26, 2022, and the Dalhousie University Annual Emergency Medical Services Research Day on October 20, 2022.

## Review

Methods

Protocol and Registration

A preliminary search of MEDLINE, the Cochrane Database of Systematic Reviews, and JBI Evidence Synthesis was conducted, and no ongoing or completed systematic or scoping reviews on the topic were identified.

The prehospital evidence-based practice (PEP) program is a knowledge translation program, maintained by the Dalhousie Department of Emergency Medicine, Division of EMS. The Division of EMS supported this rapid scoping review at the request of local EMS partners. The Cochrane Handbook for Rapid Reviews, the JBI Manual for Evidence Synthesis, and the Preferred Reporting Items for Systematic Reviews and Meta-analyses Extension for Scoping Reviews (PRISMA-ScR) checklist guided the review process and reporting respectively [17-19].

The protocol for this review was registered on Open Science Framework, on March 19, 2022; registration number: 10.17605/OSF.IO/NX4T8, and DOI: 10.17605/OSF.IO/K5P74.

Eligibility Criteria

Concept: this review explores the concept of employing clinicians in the MCC. The role of the clinician may include providing advice to incoming callers, consultation, referral, secondary triage, or peer-to-peer consultation. These roles may be conducted by paramedics, nurses, physicians, or other clinical specialists.

Context: the context involves an MCC whose role is to receive incoming 911 calls and dispatch paramedics in response to requests for these services. This review does not include urgent telecare systems whereby patients contact a clinician over the phone for general medical advice. 

Types of Information

This scoping review considered both experimental and quasi-experimental study designs including randomized controlled trials, non-randomized controlled trials, before-and-after studies, and interrupted time-series studies. In addition, analytical observational studies including prospective and retrospective cohort studies, case-control studies, and analytical cross-sectional studies were considered for inclusion, as were descriptive observational study designs (including case series, individual case reports, and descriptive cross-sectional studies) and systematic reviews that met the inclusion criteria (depending on the research question). Furthermore, text and opinion papers were also considered for inclusion. This scoping review did not consider gray literature or conference proceedings.

Restrictions

Studies published in English since the year 2000 were included. Justification for such restrictions includes the rapid nature of this review. Furthermore, we suspected that literature published before 2000 would be of limited relevance, as indicated by a large 2015 review on urgent care delivery models, which did not include any telephone triage/consultation studies conducted prior to the year 2000 [17,20].

Information Sources

This review relied on PubMed as the platform for the search for relevant studies. A single database with broad coverage for EMS literature was chosen for expediency in order to adhere to the timeline of the work request. This database had previously been tested by our team against the Embase and Cumulative Index to Nursing and Allied Health Literature (CINAHL) databases for EMS literature coverage meeting our criteria. This test yielded 95.2% coverage by PubMed. We also accepted studies suggested by team members or stakeholders, which met the inclusion criteria but were not uncovered in our PubMed search.

Search

The following search method was developed with the advice of a health sciences librarian and conducted on PubMed on February 24, 2022.

(((((Emergency medical dispatch[tiab] OR Emergency dispatch[tiab] OR Ambulance dispatch[tiab] OR 911 dispatch[tiab] OR prehospital dispatch[tiab] OR Paramedic dispatch[tiab] OR 911 calls[tiab] OR 999 calls[tiab] OR ambulance calls[tiab] OR medical communications centre[tiab] OR medical communications center[tiab] OR emergency telephone[tiab]))) Filters: English, from 2000 - 2022

Selection of Sources of Evidence

Studies were included if they investigated calls to an emergency MCC where any clinician performed a clinical role related to that call. This included but was not limited to calls for urgent or emergent care from the public where a nurse, paramedic, and/or physician performed secondary triage or advice. Clinician-to-clinician advice calls were included if the call came through the MCC.

Studies were excluded if they investigated calls to non-emergent advice lines or telemedicine calls that did not come through the MCC. Providing pre-arrival instructions, such as instructions to initiate compressions or advice to take acetylsalicylic acid (ASA), were not considered clinical roles. A single screener (JAG) was responsible for the title and abstract screening. Inclusion at the full-text stage was conducted by one reviewer (JAG) and checked by other members of the author team.

Data Charting Process

Data were extracted from each included paper by one reviewer (JAG) and checked by another reviewer (JS) by using the data extraction tool developed by the author team. Due to the rapid nature of this review, the authors of the papers were not contacted to request missing or additional data.

Data Items

The data extracted included specific details about the participants, clinical roles, study methods, key findings, and outcomes relevant to the review question.

Critical Appraisal of Individual Studies

Quality of evidence and direction of evidence evaluations were conducted using the PEP levels of evidence (LOE) and direction of evidence (DOE) scales [21]. DOE was used to indicate whether the study supported the application of the clinical role within their setting. When support was not indicated, the direction was evaluated as neutral. The PEP program LOE scale and DOE scale are summarized below (Tables 1, 2).

**Table 1:** PEP program levels of evidence scale PEP: prehospital evidence-based practice

Levels of evidence scale	
Level I	Evidence obtained from adequately powered and well-designed randomized controlled trials (RCTs) on live human participants, systematic reviews that predominantly contain RCTs, and meta-analyses
Level II	Evidence obtained from adequately powered non-randomized studies with a comparison group of live human participants, or systematic reviews/meta-analyses of non-randomized studies with a comparison group. Prospective or retrospective registry-type studies in which comparisons are made; cohort and case-control studies are included here
Level III	Evidence from studies with no randomization and no comparison group, simulation/manikin studies, and animal studies
Excluded from PEP	Opinion articles, editorials, epidemiological reports, surveys, or articles not reporting primary studies

**Table 2:** PEP program direction of evidence scale PEP: prehospital evidence-based practice

Direction of evidence scale	
Green	The results of this study are supportive of the use of this intervention
Yellow	The results of this study are neutral on the use of this intervention. The results did not show any benefit or harm associated with the intervention
Red	The results of this study oppose the use of this intervention. The results demonstrate harm or caused a negative impact
White	The results of this study are not yet evaluated

Synthesis of Results

A narrative summary was accompanied by the tabulated and/or charted results. Graphs and figures were included where appropriate*.*

Results

Selection of Sources of Evidence

Our search of PubMed yielded 1071 titles after duplicates were removed; four additional studies were added from other sources; 44 studies were reviewed at the full-text stage and 31 were included for final analysis.

**Figure 1:**
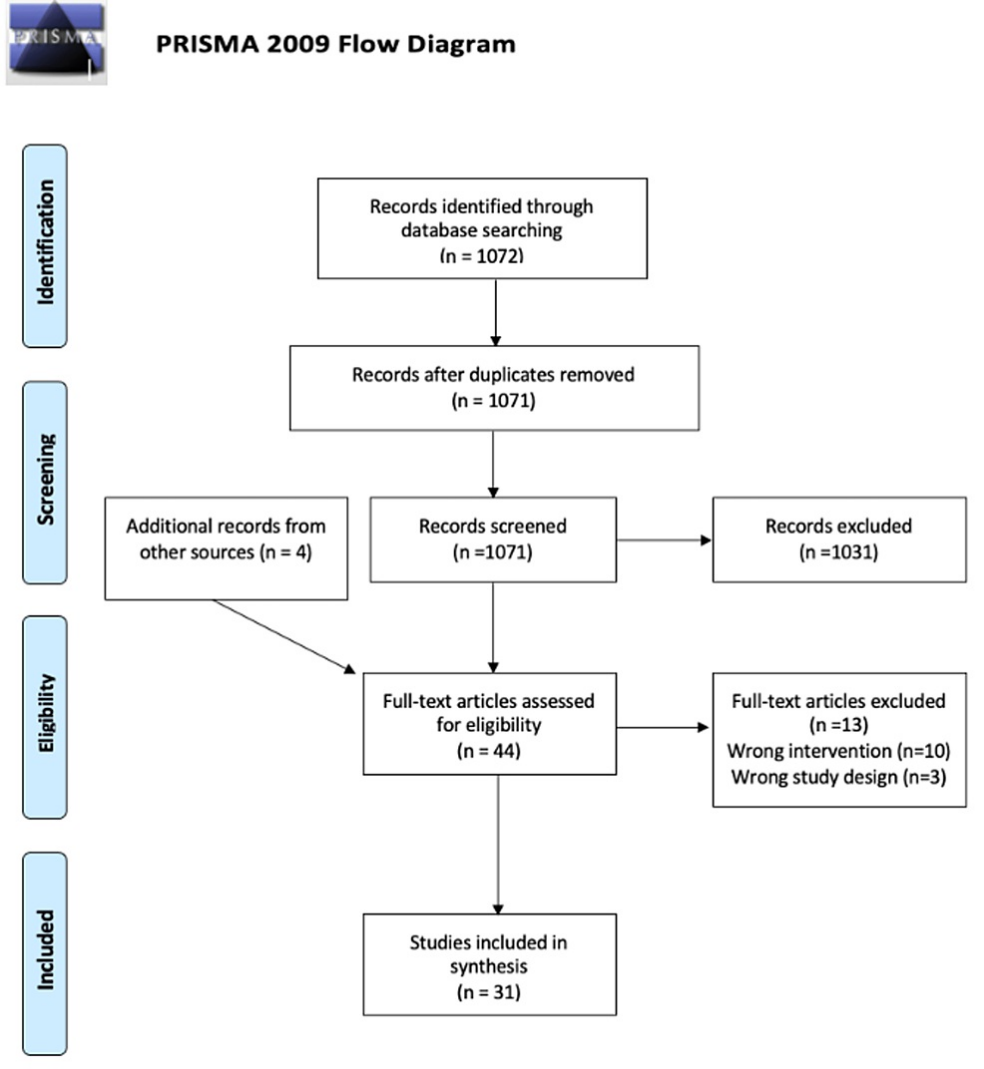
PRISMA flow diagram depicting the selection of studies PRISMA: Preferred Reporting Items for Systematic Reviews and Meta-analyses

Results and Critical Appraisal of Individual Sources of Evidence

The included studies were published from 2002 to 2022 and represent 17 countries. Studies meeting inclusion criteria consisted of level I (n=1, 3,2%), II (n=12, 38.7%), III (n=5, 16.1%) evidence, and 13 (41.9%) with designs not included in the PEP scale. The majority of the included studies were qualitative research (n=8, 25.8%) and retrospective cohorts (n=7, 22.6%). Of note, 77% percent of the studies reported a positive impact of the clinical role. Six studies did not report data demonstrating support or did not report outcomes related to success, and one found that nursing staff performed more poorly than non-clinical dispatchers by under-triaging priority (Tables 3, 4) [22].

**Table 3:** Study characteristics *N/A refers to studies that are not typically graded in the PEP LOE; **We provide the country name as reported by the study - this may include instances when the location was reported as the United Kingdom without more specifics LOE: levels of evidence; DOE: direction of evidence; PEP: prehospital evidence-based practice; RCT: randomized controlled trial

Author/year	Study design	LOE	DOE for clinical role	Location**	
					Dale et al., 2003 [23]	RCT	I	Supportive	England	
Chappuis et al., 2021 [24]	Prospective cohort	II	Supportive	Switzerland	
Eastwood et al., 2015 [25]	Systematic review	II	Supportive	United States, England, Wales	
Eastwood 2018 et al., [26]	Retrospective cohort	II	Supportive	Australia	
Eastwood 2019 et al., [27]	Retrospective cohort	II	Supportive	Australia	
Eastwood 2020 et al., [28]	Retrospective cohort	II	Supportive	Australia	
Infinger et al., 2013 [29]	Retrospective cohort	II	Supportive	United States	
Larribau et al., 2020 [30]	Prospective cohort	II	Neutral	Switzerland	
Leopardi and Sommacampagna, 2013 [31]	Retrospective cohort	II	Supportive	Italy	
Montandon et al., 2019 [32]	Systematic review	II	Supportive	Canada, United States, Japan, Croatia, England, Wales, South Korea, Norway, Finland, France, Sweden, Italy, Portugal	
Spangler et al., 2020 [33]	Prospective cohort	II	Supportive	Sweden	
Studnek et al., 2012 [34]	Retrospective cohort	II	Supportive	United States	
Torlén Wennlund et al., 2022 [22]	Retrospective cohort	II	Opposed	Sweden	
Crowther et al., 2009 [35]	Descriptive	III	Supportive	Wales	
Dale et al., 2004 [36]	Case series	III	Supportive	England	
Eastwood et al., 2016 [37]	Descriptive	III	Supportive	Australia	
O'Cathain et al., 2003 [38]	Descriptive/simulation	III	Neutral	UK	
Sakurai et al., 2021 [39]	Descriptive	III	Neutral	Japan	
Armour et al., 2022 [40]	Qualitative research	N/A*	Supportive	Canada	
Dib et al., 2006 [41]	Qualitative research	N/A*	Supportive	Netherlands	
Ek and Svedlund, 2015 [42]	Qualitative research	N/A*	Supportive	Sweden	
Foex and Walter, 2002 [43]	Text and opinion	N/A*	Supportive	France	
Forslund et al., 2006 [44]	Qualitative research	N/A*	Supportive	Sweden	
Holmström et al., 2020 [45]	Qualitative research	N/A*	Supportive	Sweden	
Holmström et al., 2021 [46]	Qualitative research	N/A*	Neutral	Sweden	
Holmström et al., 2021 [47]	Qualitative research	N/A*	Neutral	Sweden	
Jensen et al., 2022 [7]	Text and opinion	N/A*	Supportive	Canada	
Kaminsky et al., 2021 [48]	Qualitative research	N/A*	Neutral	Sweden	
Snooks et al., 2002 [49]	Literature review	N/A*	Supportive	United States, UK	
Sporer 2017 [50]	Text and opinion	N/A*	Supportive	United States, England, Denmark	
Turner 2006 [51]	Other	N/A*	Supportive	UK	

**Table 4:** Clinicians and roles within the MCC MCC: medical communications centre; EMT: emergency medical technician

Author/ year	Clinician				Clinical role							
	Paramedic	Nurse	Physician	EMT-intermediate	Triage	Advice	Referral	Peer-to-peer consult	EMD support	Dispatch	System navigation	Call-taking
Dale et al., 2003 [23]	X	X						X				
Chappuis et al., 2021 [24]	X	X								X		X
Eastwood et al., 2015 [25]	X			X	X	X	X					
Eastwood et al., 2018 [26]	X	X			X	X	X					
Eastwood et al., 2019 [27]	X	X			X	X						
Eastwood et al., 2020 [28]	X	X			X					X		
Infinger et al., 2013 [29]		X			X	X						
Larribau et al., 2020 [30]	X	X			X	X						
Leopardi and Sommacampagna, 2013 [31]		X			X							
Montandon et al., 2019 [32]		X	X		X	X	X			X		
Spangler et al., 2020 [33]		X			X	X				X		
Studnek et al., 2012 [34]		X			X	X				X		
Torlén Wennlund et al., 2022 [22]		X			X	X				X		
Crowther et al., 2009 [35]		X			X				X			
Dale et al., 2004 [36]	X	X			X							
Eastwood et al., 2016 [37]	X	X			X							
O'Cathain et al., 2003 [38]		X			X		X					
Sakurai et al., 2021 [39]		X			X	X						
Armour et al., 2022 [40]	X							X			X	
Dib et al., 2006 [41]		X			X	X	X			X		
Ek and Svedlund, 2015 [42]		X			X					X		
Foex and Walter, 2002 [43]			X		X					X		
Forslund et al., 2006 [44]		X			X					X		
Holmström et al., 2020 [45]		X			X	X	X					
Holmström et al., 2021 [46]		X			X	X						
Holmström et al., 2021 [47]		X			X		X					
Jensen et al., 2022 [7]	X		X		X		X					
Kaminsky et al., 2021 [48]		X			X	X	X			X		
Snooks et al., 2002 [49]	X	X			X	X						
Sporer 2017 [50]	X	X	X		X				X	X		
Turner 2006 [51]		X				X	X					

Synthesis of results

Clinical Roles

**Figure 2:**
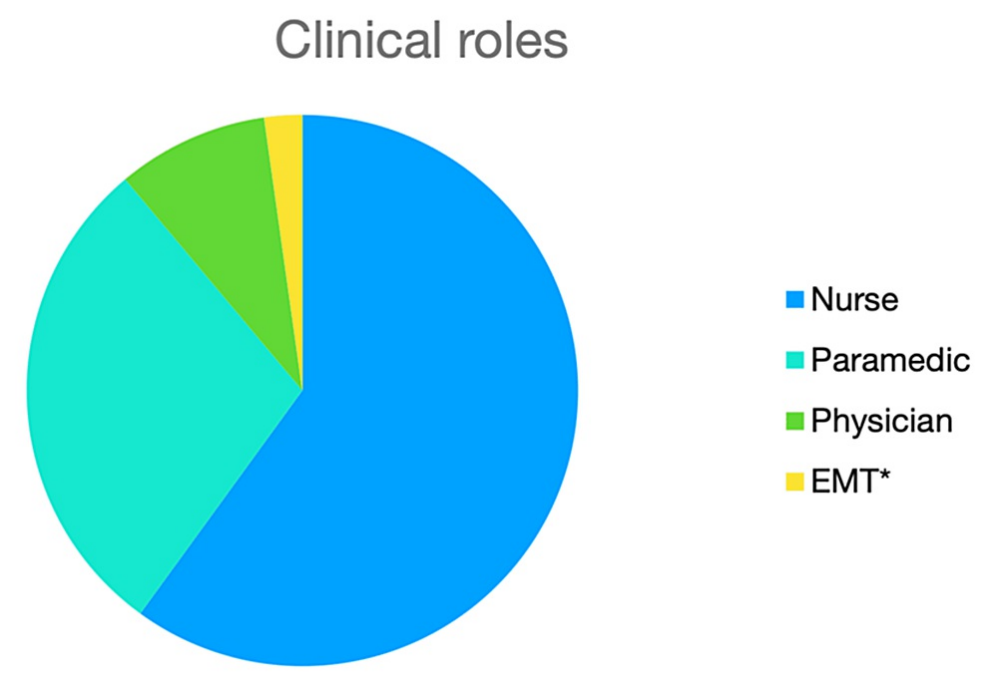
Clinicians employed within the MCC MCC: medical communications centre; EMT: emergency medical technician

The majority of the included studies reported systems that employ nurses in the MCC (n=27, 87.1%). Thirteen studies (41.9%) reported on the inclusion of paramedics in the MCC, and four (12.9%) reported physician involvement (Figure 2). The use of a clinician in the MCC was supported by the study findings in 81% of the studies (n=25). Other studies had neutral results, or it was not possible to distinguish if the study supported the inclusion of clinicians in the MCC (n=5, 16%). One Swedish study in 2022 found that nurse involvement decreased the precision of dispatch accuracy when compared to EMD-only dispatching [22]. Some studies made it a point to report the years of experience of the clinician. The reported requirement ranged from five to seven years. The Dutch MCC nursing staff required emergency medical services (EMS) experience and advanced life support (ALS) or critical care background (Figure 2) [42].

**Figure 3:**
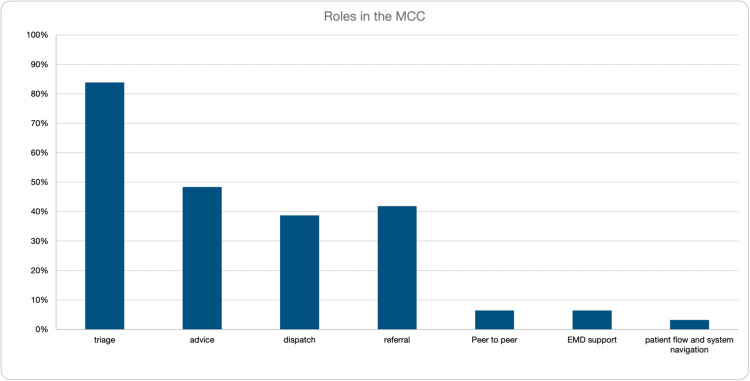
Clinical roles within the MCC MCC: medical communications centre

The clinical roles consisted of triage (n=26, 83.9%), advice (n=15, 48.4%), dispatch (n=12, 38.7%), referral to non-emergency care (n=13, 41.9.5%), peer-to-peer consulting (n=2, 6.5%), EMD support (n=2, 6.5%), and patient flow/navigation (n=1, 3.2%) (Figure 3).

MCC Roles

Some strategies dispatched ambulances and provided clinician advice/triage. Others used a dispatch priority system to direct low-acuity calls to a clinician for secondary triage, advice, or referral without the immediate dispatch of an EMS crew. The most commonly employed dispatch priority system was the Medical Priority Dispatch System (MPDS) [52]. The triage role was typically a secondary triage to determine which callers could benefit from an alternative disposition to an emergency dispatch of an ambulance. When the clinician performed dispatch, they could be dispatching an emergency response by an ambulance crew or, in some cases, a mobile physician or mobile ICU.

In systems where advice was provided, the advice could include self-care or advice to self-transport/taxi or non-emergency care. Referral options included general practitioner (GP), psychiatric care, geriatric teams, poison-control, urgent care nurse advice lines, pharmacist care, or other outreach programs. In a study reporting on the French system, some alternatives included the dispatch of a GP, an urgent response by private ambulance, an emergency medical technician (EMT), or a mobile ICU [43].

Efficacy

It was common for the clinician role to contribute to decreased ambulance use. The 2015 systematic review by Eastwood et al. reports “at least 50% of patients were diverted away from an ambulance dispatch with 31% categorized to self/home care [25].” The 2017 review by Sporer reported that 8-12% of calls were redirected [50]. A 2013 American study reported that 19.8% of patients were transferred to the nurse advice line and 12.3% received no ambulance response [29].

Safety

Few studies have reported on safety-related outcomes. In a 2004 case series of paramedic and nurse secondary triage in the United Kingdom, 96.7% of the decisions were supported by an expert review panel; the other decisions were not deemed to be life-threatening [36]. The Eastwood review and the Turner evaluation have reported the incidence of adverse events to be rare [25,51].

Discussion

While we did not set out to address non-urgent callers to the emergency MCC, we found that the majority of literature focuses on the secondary triage of non-urgent requests for care. This triage often involves advising the caller or referring to a more appropriate resource than an emergency response by a paramedic/ambulance crew.

There was considerable overlap in our findings, and a systematic review by Montandon et al. in 2019 specifically addressed the telephone triage [32]. They concluded, as we do, that prehospital telephone triage is employed globally, enabling agility and efficacy in prehospital care. Our present review goes further to describe clinical roles beyond telephone triage to other roles that may add to that agility. Some of the roles that we describe such as referral to community resources, advice lines, or self-care instructions are documented in a 2015 scoping review by Jensen et al. on alternatives to ambulance transport. They endorsed the idea that transport by ambulance cannot be solely based on the assumed necessity for care at an emergency department [3,53].

An advantage of clinical roles within an MCC may lie in support and collaboration with on-scene crews. In the past, there has been conflicting evidence on paramedic-determined non-conveyance. For example, a 2009 meta-analysis by Brown et al. found scant data in the literature on the question about which two studies in the review contributed to the aggregate conclusion of low predictive value for paramedic-determined non-transport [53]. This question has been addressed in research, and a more recent 2016 literature review found that there remains insufficient evidence to suggest that on-scene paramedics can safely determine the need for conveyance alone [54]. More research into paramedic-supported non-conveyance is required as we uncovered limited evidence for peer-to-peer on-scene support roles.

The roles we describe in our review go beyond the traditional paramedic role and include clinicians with extra training. Our review highlights the safety of clinical roles within the MCC to perform roles that may provide alternatives to traditional transport. However, it is critical to consider that many of our included studies come from highly progressive systems where entry to practice may include baccalaureate training for both paramedics and nurses [55-57].

There is a critical need to study the efficacy of clinical roles on both patient- and system-related outcomes. A thorough and deliberate investigation into the safety of alternatives to transport, including non-emergency response, referral, and self-care for non-urgent requests is required.

Limitations

The rapid review nature of this study lends to some inherent limitations. Primarily, the review was limited to a single database. We may have missed some studies that could have further informed our review. We chose PubMed for its comprehensiveness in the area of EMS, but including other recommended databases, such as Embase, would have enhanced the scope of our review. However, as mentioned earlier, PubMed covers the vast majority of EMS literature. Our search was guided by a health sciences librarian but not constructed by them and nor was our search peer-reviewed. These shortcomings were due to the time constraints put on the review. We also did not dual-screen the recommended 20% of titles. This may have introduced an element of selection bias to some extent.

## Conclusions

There is a wide range of literature reporting on clinical roles integrated within MCCs. MCC nurses, physicians, and paramedics assist substantively with triage, advice, and referrals to safely match resources to patient needs, with or without the requirement for ambulance dispatch. Clinical roles within the MCC may prove to be a cost-effective and satisfactory additional resource for patient navigation.
